# PD‐L1 and beyond: Immuno‐oncology in cytopathology

**DOI:** 10.1111/cyt.12982

**Published:** 2021-05-06

**Authors:** Antonino Iaccarino, Maria Salatiello, Ilaria Migliatico, Caterina De Luca, Gianluca Gragnano, Maria Russo, Claudio Bellevicine, Umberto Malapelle, Giancarlo Troncone, Elena Vigliar

**Affiliations:** ^1^ Department of Public Health University of Naples Federico II Naples Italy

**Keywords:** cytopathology, immune oncology, immunotherapy, PD‐L1

## Abstract

Over the past decade, immunotherapy has emerged as one of the most promising cancer treatments. Several monoclonal antibodies targeting the programmed death 1 (PD‐1)/ programmed death ligand‐1 (PD‐L1) pathway have been integrated into standard‐of‐care treatments for a wide range of cancer types. Although all the available PD‐L1 immunohistochemistry (IHC) assays have been developed on formalin‐fixed histological specimens, a growing body of research has recently suggested the feasibility of PD‐L1 testing on cytological samples. Although promising results have been reported, several important issues still need to be addressed. Among these are pre‐analytical issues, cyto‐hystological correlation, and inter‐observer agreement. This review will briefly summarise the knowledge gaps and future directions of cytopathology in the immuno‐oncology scenario.

## Abstract 2

Programmed death ligand‐1 (PD‐L1) assays have been developed on formalin‐fixed histological specimens, and a growing body of research is being dedicated to confirming the feasibility of applying these assays to cytological samples. Promising results have been reported but several important issues still need to be addressed. This review summarizes the current status, knowledge gaps and future directions of cytopathology in the context of immuno‐oncology.

## 1 INTRODUCTION

Over the past decade, immunotherapy, particularly the clinical development of immune‐checkpoint inhibitors (ICIs), has emerged as one of the most promising cancer treatments. To date, monoclonal antibodies targeting the Programmed Death 1 (PD‐1)/ Programmed Death Ligand‐1 (PD‐L1) axis have been integrated into standard treatments for a wide range of cancer types.^1^, ^2^ Despite having proven effective, ICI treatments seem to work only for a subset of patients. Not surprisingly, the identification of new predictive biomarkers for targeted treatments has become a major goal of immuno‐oncology.

In June 2020, the FDA approved pembrolizumab for the treatment of adult and pediatric patients with unresectable or metastatic solid tumour mutational burden‐high (TMB‐H) (greater than or equal to 10 mutations/megabase [mut/Mb]).^3^ At the time PD‐L1, evaluated by immunohistochemistry (IHC), was the only predictive biomarker available for PD‐L1/PD1 immunotherapy.^4^

Recently, numerous articles have been published on the topic of PD‐L1 assays, addressing factors such as clone harmonization, analytical validation, and scoring reproducibility issues.^5^, ^6^, ^7^, ^8^, ^9^, ^10^, ^11^, ^12^, ^13^, ^14^, ^15^, ^16^, ^17^ However, most of these studies involve only patients with available satisfactory formalin‐fixed paraffin‐embedded (FFPE) tissue specimens. Unfortunately, clinical research does not always reflect routine clinical practice. Indeed, PDL‐1 testing of collected tissue specimens may often be unworkable, primarily because tissue biopsies from advanced cancer patients, including those from non‐small cell lung cancer (NSCLC), are highly challenging if not impossible to obtain. Consequently, cytopathologists have no choice but to resort to cytological samples for both morphological characterisation and predictive testing. It is in this context that molecular cytopathology has emerged as a major player in diagnostic and predictive pathology. Indeed, the growing popularity of molecular cytopathology stems from the fact that most molecular tests are highly versatile and can, therefore, be applied to a wide range of cytological preparations. However, the feasibility of PD‐L1 IHC evaluation on cytological specimens still warrants thorough investigation. In fact, as of today, the commercially available PD‐L1 assays have never been validated on cytological samples.^18^ Nonetheless, since both immunostaining and predictive testing are routinely performed in cytopathology practice, pathologists have been exploring the feasibility and reliability of assessing PD‐L1 expression in cytological samples.

In this review, we will briefly summarise the knowledge gaps and future directions of cytopathology in the immuno‐oncology scenario.

## 2 PRE‐ANALYTIC ISSUES: DOES THE SAMPLE TYPE MATTER?

Several types of cytological samples are used in routine practice. However, being characterized by distinct pre‐analytical issues, each specimen should be considered as a separate entity. In particular, the common reluctance to use cytological samples for PD‐L1 evaluation primarily stems from the notion that alcohol‐based fixatives might compromise IHC staining.^6^, ^19^, ^20^ Consequently, since PD‐L1 IHC procedures have been validated only on FFPE samples, formalin‐fixed cell block (CB) preparations are generally recommended. However, not all CBs are processed in the same way. Indeed, CB preparatory techniques may vary significantly depending on several factors, that is, the choice of the fluid medium used for the FNA needle rinse (formalin, saline or alcohol‐based fixatives followed by formalin post‐fixation), the fixation time, and the method of concentration.^19^, ^21^, ^22^, ^23^, ^24^ Despite the lack of standardized preparation protocols, several lines of evidence have demonstrated that the type of fixative does not affect PD‐L1 staining. In fact, Wang et al^21^ observed that fixation with formalin only, methanol/alcohol only, or both did not affect PD‐L1 expression. Moreover, Gosney et al^25^ indicated that paired CBs fixed in either alcohol‐based solutions (CytoRich Red or CytoLyt) or neutral buffered formalin (NBF) yielded concordant PD‐L1 expression. Likewise, Lou et al^26^ observed that specimen prefixation with CytoLyt had only a negligible impact on PD‐L1 IHC staining. Table 1 presents a summary of the literature on the effects of different types of fixatives, except formalin, on PD‐L1 evaluation.

**TABLE 1:** Summary of available literature assessing the effect of different fixation type, other than formalin, on PD‐L1 evaluation

Authors (ref.)	Sample type	Preparation type	No.	Fixatives/preservatives	Antibody clone
Lloyd et al^19^	Cell lines	CB	nr	PreservCyt CytoLyt Roswell Park Memorial Institute (RPMI) cell culture media Saline	28‐8
Wang et al^21^	FNA, fluids, BAL	CB	261	Methanol/alcohol only Formalin and methanol/alcohol	22C3
Gosney et al^25^	EBUS	CB	50	CytoRich Red CytoLyt	22C3
Lou et al^26^	Fluids, EBUS‐TBNA	CB	52	CytoLyt	22C3
Jain et al^27^	Bronchial brushing/washing	LBC	26	CytoRich Red	SP263
Capizzi et al^28^	FNA	Smears	49	MicroFix spray	SP263
Lozano et al^29^	FNA	Smears	62	Alcohol	22C3, SP263
Noll et al^30^	FNA	Smears	41	Alcohol	22C3

Abbreviations: BAL, broncho‐alveolar lavage; CB, cell‐block; EBUS‐TBNA, endobronchial ultrasound‐guided transbronchial needle aspiration; FNA, fine needle aspiration; LBC, liquid‐based cytology; No., number of samples; nr, not reported; ref, reference number.

**FIGURE 1:**
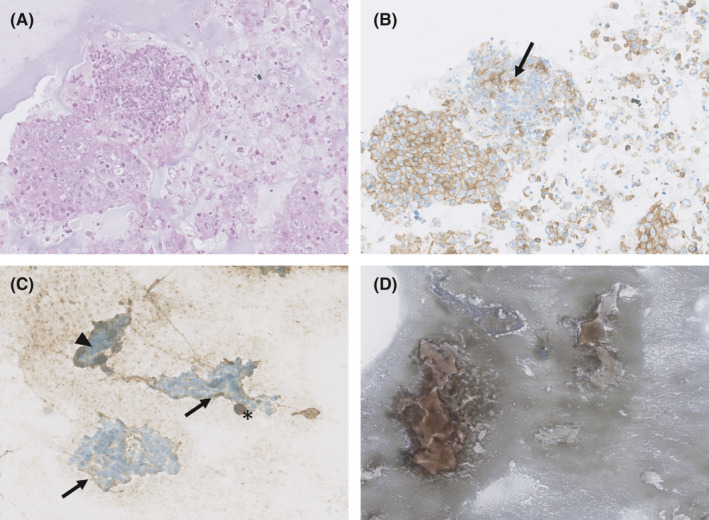
(A) Hematoxylin and eosin‐stained cell block section (original magnification 20×) and corresponding PD‐L1–stained cell block section (B): a circumferential pattern of membrane staining in neoplastic cells was observed. PD‐L1 positive lymphocytes showed indistinguishable membrane and cytoplasmic staining, due to a high nuclear to cytoplasmic ratio (arrow). (C) PD‐L1–stained ethanol‐fixed direct smear: a partial circumferential pattern of membrane staining in neoplastic cells was observed (arrows). The presence of false‐positive staining in three‐dimensional cell groups entrapping reagents (arrowhead) and staining in inflammatory cells (asterisk, histiocyte) can lead to an overestimation of the PD‐L1 expression (original magnification 20×). (D) PD‐L1–stained ethanol‐fixed direct smear: high amount of non‐specific staining of extracellular mucus (original magnification 2.5×)

Evidence that the type of fixative does not compromise PD‐L1 staining is also confirmed by studies assessing the feasibility of using “traditional” non‐formalin fixed cytological preparations, including direct smears or liquid‐based cytology specimens (LBC) for PD‐L1 IHC testing.^27^, ^28^, ^29^, ^30^ Indeed, although some studies have indicated that FFPE samples and corresponding non‐formalin fixed cytological smears show a good concordance rate of PD‐L1 expression, these preparations may lead to cytopathological misinterpretation. For instance, the presence of a non‐specific staining of neoplastic cell cytoplasms, extracellular mucus, background cellular debris,^31^ and inflammatory cells may result in an overestimation of PD‐L1 expression on direct smears. Moreover, appreciation of true membranous staining, which is perceived as distinct from cytoplasmic staining, and the presence of false‐positive staining in large three‐dimensional cell groups entrapping reagents, may also lead to a misinterpretation of PD‐L1 expression on direct cytological smears.^32^ (Figure 1).

## 3 CYTO‐HISTOLOGICAL CORRELATION

To assess whether cytological samples are as reliable as histological samples for PD‐L1 testing, several authors have intensively investigated the concordance rates between matched cytological and histological samples. Indeed, several research groups have evaluated both cytological and histological samples in terms of adequacy rates and PD‐L1 expression levels. More specifically, the adequacy criteria state that a sample must contain a minimum of 100 viable tumour cells to be eligible for quantification of PD‐ L1 expression. It is worth noting that the literature has shown that the adequacy rate of cytological samples is generally higher than 80%^21^, ^42^ and only occasionally lower than 70%^44^ (Table 2). These data are remarkable if one considers the great difficulty of obtaining a sufficient number of tumour cells in small biopsies.^38^ It is also of note that rapid on‐site evaluation (ROSE) can be used to assess specimen adequacy and possibly improve CB quality in terms of tumour cellularity.^45^ However, conclusive evidence regarding the use of ROSE on downstream ancillary testing outcomes is still lacking.^46^, ^47^ Under this scenario, cytopathologists play a key role, not only in carrying out on‐site evaluation of cytological material, but also in ensuring the proper triage of available material.

**TABLE 2:** Summary of available literature assessing adequacy rate of PD‐L1 evaluation on cytological samples

Authors (ref.)	Sample type	Preparation type	No.	Adequacy rate %
Wang et al^21^	FNA, fluids, BAL	CB	371	92
Noll et al^30^	FNA	CB/smears	41	92.6/90.2
Zou et al^33^	Fluids	CB	124	91.9
Torous et al^34^	TBNA, pleural effusion, bronchial washing	CB	94	93.6
Evans et al^35^	nr	CB	2276	84
Bubendorf et al^36^	Fluids, washing, brushing, FNA, ex vivo FNA	CB	165	86.6
Vigliar et al^37^	nr	CB/smears/LBC	48	85.4
Heymann et al^38^	FNA, fluids	CB	40	90
Mei et al^39^	Fluids, FNA	CB	100	96
Skov et al^41^	nr	CB	86	80.3
Stoy et al^41^	TBNA	CB	22	90.9
Dong et al^42^	FNA, brushing	CB	112	70.5
Kravstov et al^43^	nr	CB	75	84
Hendry et al^44^	Bronchial brushing, FNA	CB	60	50

Abbreviations: BAL, broncho‐alveolar lavage; CB, cell‐block; FNA, fine needle aspiration; LBC, liquid‐based cytology; No., number of samples; nr, not reported; ref, reference number; TBNA, transbronchial needle aspiration.

As for PD‐L1 evaluation, since 2017 several single institutional studies have reported comparable PD‐L1 expression on matched cytological and histological (small biopsy/surgical resection) specimens.^21^, ^47^, ^48^, ^49^, ^50^, ^51^ In light of these findings, in a systemic review, Gosney et al^52^ painstakingly evaluated the concordance rate of PD‐L1 staining in matched histological and cytological samples from patients with advanced NSCLC. Based on a total of 428 paired specimens collected across nine studies, the authors reported an overall concordance rate of 88.3% at a clinically relevant tumour proportion score (TPS) cut‐off greater than 1% and of 89.7% for specimens with TPS greater than or equal to 50%. Interestingly, these values closely reflect sample heterogeneity in real‐life cytology practice. In fact, the review evaluated data from both CBs and direct smears obtained from different sampling types (endobronchial ultrasound, computed tomography and ultrasound guided FNA, washing, brushing and fluid collection). Moreover, it also examined different PD‐L1 antibody clones (22C3 [Dako] and SP263 [Ventana]) using both pharmDx assays and laboratory developed tests (LDTs). The clear concordant results confirm once again the reliability of using cytological material for PD‐L1 evaluation. Interestingly, Dong et al's study^42^ pointed out that CBs with higher cellularity show better agreement scores between cytology and histology. Indeed, PD‐L1 expression levels in resected specimens were nearly equivalent to those in CBs with abundant cellularity (greater than 400 cells). Altogether, these studies clearly indicate that cytological materials constitute a reliable source for PD‐L1 evaluation in NSCLC patients.

## 4 INTEROBSERVER AGREEMENT

Cytopathologists should take into account interobserver variability rates before deeming cytological specimens suitable for PD‐L1 assessment. However, as of today, data on interobserver agreement are still limited to a few studies involving varying numbers of pathologists and analyzed samples. Overall, though, reproducibility has been remarkable. For example, Russell‐Goldman et al^53^ reported a high interobserver agreement (intraclass correlation coefficient, ICC equal to 0.96) between two pathologists who evaluated 56 cytological specimens. Similarly, Gagnè et al^54^ reported substantial or almost perfect interobserver agreement rates (Fleiss' kappa, k equal to 0.74‐0.82) among four pathologists who evaluated 46 CBs. Consistently, the Blueprint (BP) PD‐L1 Immunohistochemistry Comparability Project phase 2^6^ reported a good ICC at all cutoff levels (k equal to 0.60‐0.80), for both glass (0.78) and digital (0.85) slides among 24 pathologists who analyzed 22 CBs. More recently, quite similar interobserver agreement rates were reported by Sinclair et al^55^ (k equal to 0.74) and Kravsotv et al^43^ (k equal to 0.66) (Table 3). Despite such encouraging agreement rates, some studies have highlighted the fact that that variability among observers is generally more pronounced in cytological samples than in biopsies and surgical specimens,^6^, ^56^ suggesting that the interpretation of PD‐L1 in cytological samples is more challenging. The main difficulties arise primarily from the presence of background aspecific staining and the difficulty of differentiating tumour cells from benign ones, including macrophages, especially in cases presenting discohesive cells. Moreover, these pitfalls are more pronounced in traditional, non‐formalin fixed cytological preparations, for which data on interobserver agreement are still lacking. For this reason, deciding whether a cytological sample is appropriate for PD‐L 1 IHC assessment requires considerable expertise and specialized training.^43^, ^56^

**TABLE 3:** Summary of literature studies assessing interobserver agreement for PD‐L1 scoring on cytological specimens

Authors (ref.)	Preparation type	No.	Number of pathologists	Antibody clone	Statistical test	Interobserver agreement
Tsao et al^6^	CB	22	24	22C3, 28‐8, SP142, SP263, 73‐10	ICC Fleiss's kappa	ICC =0.78‐0.85 k = 0.6‐0.85
Kravstov et al^43^	CB	50	3	22C3	Fleiss's kappa	k = 0.66
Russel‐Goldman et al^53^	CB	56	2	E1L3N	ICC	0.96
Gagnè et al^54^	CB	46	4	SP263, 28‐8	Fleiss's kappa	k = 0.74 to 0.82
Sinclair et al^55^	CB	86	5	22C3	Fleiss's kappa Cohen's kappa	0.74‐0.79 0.49‐0.83 to 0.63‐0.90

Abbreviations: CB, cell block; ICC, intraclass correlation coefficient; No., number of samples; ref, reference number.

## 5 GUIDELINES

The literature has clearly established that cytology specimens (smears, CBs, LBC) are valuable sources for ancillary techniques, provided that careful validation of the samples is carried out.^57^ Consequently, recommendations for proper management of cytological material have been included in biomarker testing guidelines for patient selection in immuno‐oncology. For example, the Canadian Association of Pathologists‐Association Canadienne Des Pathologistes (CAP‐ACP) recommends that FDA‐approved or CE‐marked PD‐L1 IHC kits, validated for FFPE samples, be used for cytology samples only if they are processed according to the pre‐analytical conditions provided by the kit and the readout is compatible with the type of cytology samples.^58^ For NSCLC cases, the International Association for the Study of Lung Cancer Pathology Committee (IASCL) requires that protocols for cytological materials be fully validated and submitted to quality‐control measures. Thus, it stands to reason that validation processes ought to be carried out separately for any type of cytological preparation.^18^

## 6 FUTURE PERSPECTIVES

Current advances in both digital image analysis (DIA) technologies and multiplex immunofluorescence (IF)/IHC could be a powerful strategy for PD‐L1 assessment. In fact, the application of a high throughput image analysis pipeline to multiplex IF or IHC to assess PD‐L1, the epithelial cell marker cytokeratin, the macrophage marker CD68, and the T‐cell marker CD8 has been shown to yield a high diagnostic level of confidence in the identification of specific cell types co‐expressing PD‐L1. Therefore, a multiplex approach may enable cytopathologists to refine PD‐L1 scores in neoplastic cells, especially in cases close to clinical thresholds. Nonetheless, cytological samples pose practical issues due to a lack of tissue architecture. Therefore, further investigations are warranted to investigate the diagnostic accuracy of the PD‐L1 multiplex image analysis on cytological specimens.^59^, ^60^, ^61^, ^62^

It is widely known that predicting ICI therapy outcome on the basis of a single biomarker, such as PD‐L1, is far from perfect. Therefore, promising predictive biomarkers are currently under investigation, including TMB, defined as the total number of somatic mutations per tumour genome. Although most of the data on TMB are derived from the evaluation of FFPE histological samples,^63^, ^64^ some authors have provided preliminary results on the feasibility of assessing TMB on cytological material. For example, Pepe et al^65^ recently demonstrated the technical feasibility of assessing TMB on FFPE CBs in a pilot study evaluating 16 paired histological and CB samples from eight NSCLC patients. Interestingly, Alborelli et al,^66^ who compared TMB values in matched FFPE and cytological specimens, demonstrated that cytological smears provide even more consistent TMB values than their histological counterparts. Therefore, considering the high quality of DNA and lack of formalin‐fixation induced artifacts, the authors concluded that ethanol‐fixed cytological specimens allow a more robust TMB estimation than histological samples.

However, immunotherapy outcomes may significantly vary among patients, regardless of PD‐L1 expression and TMB values. Thus, major efforts are being made to identify co‐occurring mutations. For example, Marinelli et al^67^ identified four genes (*KEAP1, PBRM1, SMARCA4* and *STK11*) that potentially reduce the efficacy of immunotherapy in patients with lung adenocarcinoma. Thus, the dynamic nature of immuno‐oncology highlights the relevance of managing cytological materials appropriately to maximise their use for comprehensive predictive testing.

Finally, in addition to tumour cells, the tumour microenvironment (TME) and its dynamic reshaping have emerged as major players in cancer progression and treatment outcomes. The importance of this line of research is reflected in the recent development of ultra‐fast cycling for multiplexed cellular fluorescence imaging for the analysis of single cell populations, such as those analyzable in cytological samples.^68^ This new approach could break new ground in the evaluation of immunological dynamics by exploiting the ability of cytopathologists to perform serial cytological tumour sampling.^69^

**FIGURE 2:**
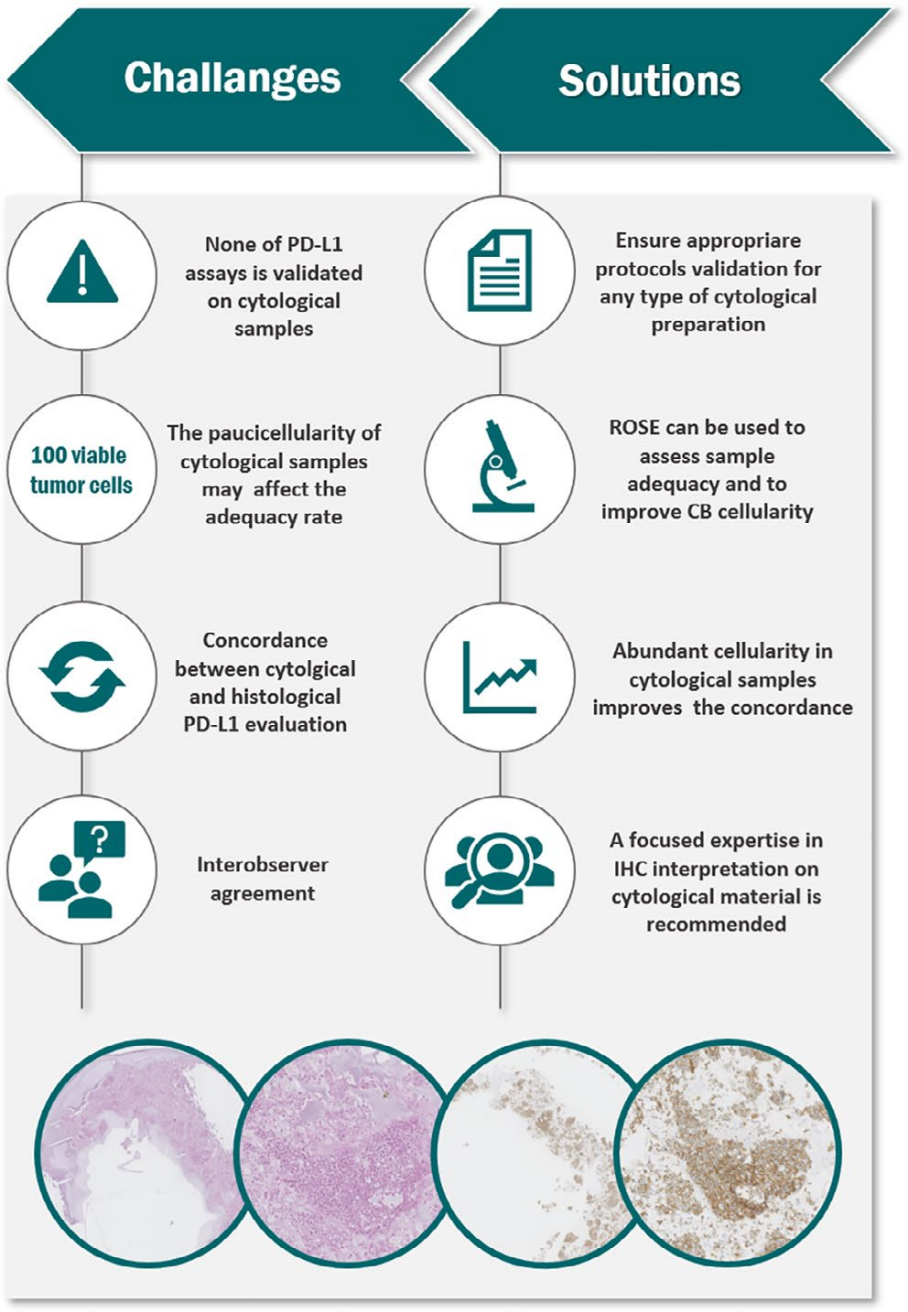
Schematic representation of challenges and solutions in PD‐L1 evaluation on cytological material. IHC, immunohistochemistry; ROSE, rapid on‐site evaluation

In conclusion, this review clearly indicates that cytological samples constitute a reliable source for PDL‐1 IHC analysis (Figure 2), as evidenced by the remarkable specimen adequacy and concordance rate seen between cytological and histological specimens. Moreover, the fact that that cytological fixatives do not compromise PD‐L1 staining further attests to the utility of cytological specimens for PD‐L1 testing in routine clinical practice. However, there are few challenges which still need to be addressed. In particular, training programs should be provided to ensure adequacy assessment and proper sample management, and preparation protocols must be validated and standardized across individual laboratories. Moreover, the value of dedicated expertise in PD‐L1 interpretation in cytological samples cannot be underestimated.

Finally, although much of the new evidence regarding TMB and TME is still preliminary, we are confident that cytological samples will have great utility in precision immuno‐oncology.

## CONFLICT OF INTEREST

Umberto Malapelle received personal fees (as speaker's bureau or advisor) from Boehringer Ingelheim, AstraZeneca, Roche, MSD, Amgen and Merck, for work unrelated to the current paper. Giancarlo Troncone received personal fees (as speaker's bureau or advisor) from Roche, MSD, Pfizer and Bayer, for work unrelated to the current paper. Elena Vigliar received personal fees as advisor from Diaceutics, for work unrelated to the current study. The other authors declare no potential conflicts of interest.

## AUTHOR CONTRIBUTIONS

Antonino Iaccarino, Maria Salatiello, Giancarlo Troncone and Elena Vigliar conceived the review. All authors collected the literature data, wrote the original draft, and approved the final version of the manuscript.

## Data Availability

Data sharing not applicable to this article as no datasets were generated or analysed during the current study.
